# Online ECG Biometrics for Streaming Data with Prototypes Learning and Memory Enhancement

**DOI:** 10.3390/s25092908

**Published:** 2025-05-04

**Authors:** Kuikui Wang, Na Wang

**Affiliations:** 1 School of Computer Science and Technology, Shandong Jianzhu University, Jinan 250101, China; 2 School of Software, Shandong University, Jinan 250101, China; wangna.wn2000@gmail.com

**Keywords:** ECG biometrics, online, prototypes, memory

## Abstract

Recently, electrocardiography (ECG) has attracted significant attention in the field of biometrics, presenting a compelling alternative for biometric recognition based on physical or biological traits. Impressive application results have been achieved by existing methods, the majority of which are designed in the batch processing mode. The batch mode inherently assumes that all data can be acquired prior to training the final model and that no new data will subsequently arrive. Clearly, this assumption is unrealistic, as real-world data often arrive in a streaming fashion, meaning that they are continuously generated and transmitted. When confronted with streaming data, traditional batch-based methods require re-training on all the data once again, including both the newly arrived data and the previously trained data. Consequently, these methods lead to redundant calculations and significant expenses. To overcome this limitation, we propose a new online method for ECG biometrics that incrementally learns from streaming data. Our method updates itself with only the new arriving data, eliminating the need to retrain with both old and new data. To enhance the discriminative power of to-be-learned sample representations, we introduce two novel modules: bidirectional regression and prototype learning. Since our method does not revisit old data when new data arrive, we incorporate a memory enhancement module to mitigate the catastrophic forgetting problem caused by a lack of exposure to old data. Furthermore, we design a novel and efficient online optimization algorithm to minimize the overall loss function. Extensive experiments conducted on two widely used datasets demonstrate the effectiveness of our proposed method.

## 1. Introduction

ECG biometrics is a promising approach for individual identification and verification due to the uniqueness of electrical heart signals. Compared with conventional biometric traits, such as faces, fingerprints, and irises, ECG is easily accessible and unique in all potential users, regardless of physical or mental conditions [1]. Numerous research studies have been presented in the literature that utilize ECG for biometric recognition, and the existing methods can be broadly categorized into two types: hand-crafted feature extraction methods and non-hand-crafted feature-based approaches. Methods of the first type tackle ECG biometrics by elaborately designing various feature extraction techniques. For example, representative hand-crafted features include, but are not limited to, the following: fiducial features [2], sparse representation [3,4], wavelet features [5,6], autocorrelation (AC) features [7,8], and discrete cosine transform (DCT) [9,10]. Recently, owing to the great success of deep learning and related techniques [11,12], non-hand-crafted feature-based methods have become popular. Among them, different model architectures can be utilized, e.g., convolutional neural (CNN) networks [13,14], long short-term memory (LSTM) networks [15], and so on [16].

Although existing methods have achieved good results, they still fall short of real-world settings and are unable to handle some of the challenges presented in practical problems. Firstly, in practical application scenarios, ECG biometrics may be implemented on devices with constrained resources. These devices may lack adequate storage capacity or computing power. If storage space is inadequate, it becomes impractical to store and use all the data for training. When computing power is insufficient, the utilization of complex methods, which are built upon deep learning, will be limited, as these methods often have a large number of parameters and result in expensive computation costs. Secondly, nearly all previous methods assume that all data can be comprehensively collected at the same time, which is at odds with how data are acquired in real world. Typically, data are continuously collected, and this process results in data often referred to by researchers as “**streaming data**”. The existing methods are not designed for streaming data, as their learning mode is characterized as **batch-based**. Batch-based methods implicitly assume that all required data are available when they start training the models from scratch. When new data are collected, they cannot adjust the existing model solely based on the new data; instead, they must retrain the models using both the new and old data. This batch-based training strategy has obvious drawbacks: it lacks flexibility and cannot efficiently adapt to streaming data scenarios, leading to large redundant calculations.

Fortunately, a novel approach [17] has been proposed to address these issues, which introduces the “**online learning mode**” into the area of ECG biometrics. To the best of our knowledge, this is the first work to tackle the incremental learning problem for ECG biometrics. The online biometric setting is practical in terms of its similarity to real-world ECG biometric tasks. The online learning strategy enables models to incrementally update themselves without the necessity of revisiting all previously old data. In other words, the online learning strategy allows the model to focus more on newly collected data. Since previously trained data are not involved, this online learning strategy enables the avoidance of the issue of significant repetitive calculations. Additionally, online ECG biometrics circumvent the substantial computational requirements for both training and inference by designing a non-deep learning framework, making itself flexible and scalable.

Despite addressing several existing issues in ECG biometrics, there remain numerous challenges that the pioneering method [17] fails to address. Firstly, applying the idea of online learning to handle streaming data encounters the well-known **catastrophic forgetting** problem. Catastrophic forgetting refers to the phenomenon where, if old data are not reused for training, the updated model may forget the knowledge learned from the old data and become biased towards the new incoming data. The existing method [17] fails to give full consideration to this problem. Secondly, streaming data pose the challenge of the class-incremental problem, where new users (i.e., new categories) emerge alongside new data. To tackle this problem, the existing method [17] uses class prototypes, which are defined before learning and are independent of the data. Such prototypes imply that the existing method fails to fully learn from the data.

**Figure 1:**
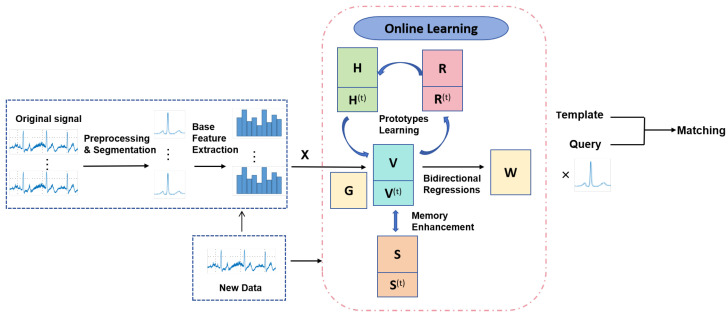
The framework of our method.

To overcome the limitations mentioned above, we propose a new framework called Online ECG Biometrics with Prototypes Learning and Memory Enhancement, and the framework is illustrated in Figure 1. As shown in Figure 1, we first preprocess the ECG signals via segmentation to generate heartbeats, and then extract the base features. Subsequently, our online learning framework transforms these base features into discriminative representations through three well-designed modules, i.e.,, bidirectional regressions, prototypes learning, and memory enhancement. Finally, the learned projection matrix is applied to extract latent representations from both query and registered samples for the matching procedure. Specifically, to address the situation that ECG data are continuously generated and transmitted, and therefore arriving in a streaming manner, our method could be incrementally trained with only new data rather than both old and new data. To obtain more discriminative representations for samples, we propose bidirectional regressions and prototypes learning modules. To alleviate the catastrophic forgetting problem, we further introduce a memory enhancement module. Finally, a novel and efficient online optimization algorithm is presented to solve the overall loss function.

The main contributions of this paper are summarized as follows.

- We propose a novel ECG biometrics method, which is designed to be in the online mode, with three well-designed modules, i.e., bidirectional regressions, prototypes learning, and memory enhancement.
- For streaming data, our online method is capable of learning discriminative representations while effectively mitigating the catastrophic forgetting problem and addressing the class-incremental problem.
- Experimental results from two datasets demonstrate that the proposed method performs better than all baselines, demonstrating the effectiveness of our method.

The remaining part of this paper is organized as follows. Section 2 briefly summarizes the related works. Section 3 details the proposed online ECG biometrics for streaming data. Section 4 outlines the experimental setup and results. Lastly, Section 5 concludes the paper with a brief summary.

## 2. Related Works

Generally speaking, ECG is a physiological signal produced by the heart’s contraction and relaxation cycles. A standard ECG primarily comprises the P wave, the QRS complex wave, and the T wave, where the peaks of P, Q, R, S, and T waves are regarded as fiducial points. ECG biometrics has gained popularity as a trend, with numerous methods being proposed, and most existing ECG biometric approaches can be categorized into two groups: hand-crafted feature-based and non-hand-crafted feature-based approaches. Depending on the type of features they utilize [18], the hand-crafted feature-based methods can be further divided into the following categories: fiducial, non-fiducial, and hybrid methods.

Fiducial methods are usually based on the morphology of the signal. They involve detecting fiducial points, such as the peaks of P, Q, R, S, and T waves, as well as amplitudes, durations, QRS complexes, angles, and slopes. On the contrary, non-fiducial methods do not use these fiducial points. For example, Biel et al. [19] have extracted 30 features based on the delineating dominant fiducial points, i.e., wave onset, durations, amplitudes, areas, and confidence. Arteaga et al. [20] leveraged eight fiducial features, including six time-based and two amplitude-based features. Barros et al. [21] utilized 3-s segments to extract QRS wave amplitudes, durations, intervals, and the standard deviations of the Q, R, and S waves.

Non-fiducial methods typically concentrate on the entire signal or on segments analyzed through a sliding window, aiming to eliminate the necessity for detecting fiducial points altogether. For example, Fatimah et al. [22] leveraged the phase transform (PT) and Fourier decomposition method (FDM) for ECG identification. Galli et al. [23] utilized Gaussian kernels to generate features from the cardiac cycle. Hejazi et al. [24] proposed a non-fiducial framework that employed the discrete wavelet transform (DWT) and kernel methods for noise reduction and feature dimensionality reduction, respectively.

Hybrid methods usually integrate characteristics from both fiducial and non-fiducial algorithms, and numerous previous works have shown success using sparse representation [25,26], matrix factorization [27,28,29], and dimensionality reduction [30,31]. For example, Huang et al. [4] proposed a unified sparse representation framework which collaboratively exploits joint and specific patterns for ECG biometric recognition. Wang et al. [28] leveraged collective matrix factorization to generate robust ECG representations by simultaneously embedding MSDF-1DMRLBP and label information. Wu et al. [32] utilized the concept of signal subspace collapsing to construct distinct biometric templates in order to identity the individual.

Recently, while deep learning techniques have garnered great success across diverse domains [33,34], they have also been employed for ECG biometric recognition. As those methods could benefit from end-to-end learning, they do not need to specifically design features; therefore, we call them non-hand-crafted feature-based approaches. Li et al. [13] designed a generic convolutional neural network approach to realize human identification. Ihsanto et al. [14] proposed a residual depthwise separable convolutional neural network for ECG authentication. Jyotishi et al. [15] designed a novel attention-based hierarchical long short-term memory (HLSTM) model to learn the biometric representations corresponding to a specific person. Rincon et al. [35] focused on utilizing a transformer neural network for biometric identification. Srivastva et al. [36] proposed an ensemble of the pre-trained ResNet and DenseNet for ECG biometric recognition.

## 3. Proposed Method

### 3.1. Notations and Problem Definition

As we introduced a novel online ECG biometrics method designed for streaming data, we demonstrated our approach within an online setting. In this scenario, samples continuously arrive in a streaming fashion. Specifically, some data may appear at one time, while other data may appear at a later time, and this situation will continue to occur. The point in time at which data appear is termed a “data round”, and the data presented in each round are called a “data chunk”. Without a loss in generality, we take the *t*-th round as an example to illustrate our method.

At the *t*-th data round, $n_{t}$ ECG signals arrive and we name them as new data. By extracting features from those ECG signals, we obtain the feature matrix of the new data ${\vec{X}}^{\left(t\right)}\in R^{d\times n_{t}}$, where *d* is the dimensionality of the extracted features. Corresponding to the newly emerging data in the current round, we refer to the data that have been previously seen as old data and denote them as ${\tilde{X}}^{\left(t\right)}$$\in R^{d\times (N_{t-1})}$. Here, $N_{t-1}$ is the number of all seen old ECG signals from the first round to the last round and $N_{t-1}=n_{1}+\cdots +n_{t-1}$. The arrow symbol ^→^ denotes matrices for the current round, while the tilde symbol ^˜^ corresponds to matrices before round *t*.

**Figure 2:**
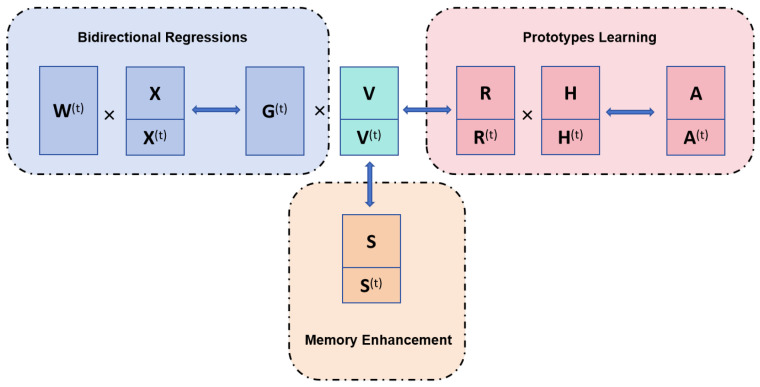
The integrated flowchart of the three modules.

The problems with online ECG biometrics can be summarized as follows: (1) We want to create a latent learning space where samples can have discriminative representations. To transform data from base features to this space, we need to create a projection matrix $W^{\left(t\right)}$. (2) At each round, our method should learn from current data that contain both extracted features ${\vec{X}}^{\left(t\right)}$ and the label information; ${\vec{X}}^{\left(t\right)}$ is obtained from the original ECG signals. The detailed methodology used in this paper is presented in Section 4. The label information can tell us to which individual each sample belongs. Furthermore, one individual’s different samples should have similar representations to each other. As mentioned above, the proposed framework is designed in the online mode, with three well-designed modules, i.e., bidirectional regressions, prototypes learning, and memory enhancement. The flowcharts for these modules are shown in Figure 2. As shown in Figure 2, all three modules contribute to learning discriminative features, and the technical details for these modules are provided in Section 3.2, Section 3.3 and Section 3.4.

### 3.2. Bidirectional Regressions

Since most existing methods typically adopt a batch-based strategy and the feature extraction procedure does not take our data into consideration, the extracted features ${\vec{X}}^{\left(t\right)}$ may be suboptimal if we do not adapt them to our task and data. Hence, we try to distill effective information from ${\vec{X}}^{\left(t\right)}$ using the following bidirectional regression module:

(1) $$\begin{array}{c} \begin{array}{cc} & O_{1}=\alpha \left({∥{\tilde{V}}^{\left(t\right)}-{\tilde{X}}^{(t)⊤}W^{\left(t\right)}∥}_{F}^{2}+{∥{\vec{V}}^{\left(t\right)}-{\vec{X}}^{(t)⊤}W^{\left(t\right)}∥}_{F}^{2}\right)+\\ & \beta ({∥{\tilde{X}}^{\left(t\right)}-G^{\left(t\right)}{\tilde{V}}^{(t)⊤}∥}_{F}^{2}+{∥{\vec{X}}^{\left(t\right)}-G^{\left(t\right)}{\vec{V}}^{(t)⊤}∥}_{F}^{2}), \end{array} \end{array}$$

where ${∥\cdot ∥}_{F}$ denotes the Frobenius norm of a matrix, α and β are trade-off parameters, $W^{\left(t\right)}$ and $G^{\left(t\right)}$ are bidirectional projection matrices, and ${\vec{V}}^{\left(t\right)}\in R^{n_{t}\times r}$ represents $n_{t}$ new samples in the to-be-learned *r*-dimensional latent space.

As a discriminative latent space could significantly improve the quality of our ECG biometrics model, it is crucial to create this latent space. Thus, we designed the bidirectional regressions shown in (1). Specifically, terms controlled by the parameter α aim to reconstruct the learned representations V from the original extracted feature X, while terms controlled by β extract the low-rank information V from the feature X.

Additionally, we use information from both old data (${\tilde{X}}^{\left(t\right)}$ and ${\tilde{V}}^{\left(t\right)}$) and new data (${\vec{X}}^{\left(t\right)}$) in (1) because we want the latent representations of the new data ${\vec{V}}^{\left(t\right)}$ to be able to learn from not only their features ${\vec{X}}^{\left(t\right)}$ but also from the already learned representations of the old data ${\tilde{V}}^{\left(t\right)}$. This design could help alleviate the catastrophic forgetting problem in an online setting and ensure better ${\vec{V}}^{\left(t\right)}$.

### 3.3. Prototypes Learning for Individuals

For ECG biometrics, it is optimal that the similarity within a class is large and the similarity between classes is low. That is to say, representations for different individuals should differ significantly, while representations for one individual should be similar. To achieve these two goals, we propose learning a unique prototype for each individual and utilizing these learned prototypes to guide the learning of representations ${\vec{V}}^{\left(t\right)}$. On the one hand, prototypes for different individuals should be dissimilar. On the other hand, the learned representations for a given individual should be as close as possible to that individual’s prototype. The proposed prototype learning module for individuals is as follows:

(2) $$\begin{array}{c} \begin{array}{cc} & O_{2}=\gamma {∥{\vec{A}}^{\left(t\right)}-{\vec{H}}^{\left(t\right)}{\vec{R}}^{(t)⊤}∥}_{F}^{2}+\theta {∥{\vec{R}}^{\left(t\right)}-{\vec{V}}^{\left(t\right)}∥}_{F}^{2}, \end{array} \end{array}$$

where matrix ${\vec{A}}^{\left(t\right)}$ is a pairwise similarity matrix between new samples, where its elements represent whether the samples belong to the same individual (1) or not (0). γ and θ are the trade-off parameters. ${\vec{H}}^{\left(t\right)}$ denotes the assigned Hadamard code matrix of new samples at round *t*. ${\vec{R}}^{\left(t\right)}\in R^{n_{t}\times r}$ is the matrix composed of prototypes corresponding to the new samples. Please note that ${\vec{H}}^{\left(t\right)}$ is pre-defined before learning and that the way in which we utilize the Hadamard code matrix is the same way utilized in [17]. Each row of prototype matrix ${\vec{R}}^{\left(t\right)}$ corresponds to a sample in data chunk *t*. Specifically, if two samples belong to the same individual, then the corresponding rows share the same prototype, which is also the prototype of this individual. With this prototype learning module, we can obtain discriminative prototypes and subsequently learn better latent representations ${\vec{V}}^{\left(t\right)}$ for samples.

### 3.4. Memory Enhancement

The proposed online learning based method can more efficiently deal with streaming data than batch-based methods, but such an advantage does not come without a cost. One noticeable issue is the catastrophic forgetting problem. Catastrophic forgetting refers to the phenomenon where, when encountering new data during continuous learning, the model tends to significantly forget the knowledge it has learned from previous old data. In this paper, we propose a novel memory enhancement module to alleviate the problem. When new data arrive in the current round, if we can update the model using both the old data from previous rounds and the new data, the issue of catastrophic forgetting can be mitigated. However, if we can store and use 100% of the data from previous rounds, it would essentially become a batch-based method. Therefore, we need to limit the amount of old data that can be used. The fewer old data we use, the lower the storage cost and computational demand for model updates, making our method more suitable for real-world scenarios.

We aim to establish a memory that efficiently stores the feature information of all encountered individuals while minimizing its size. A straightforward and effective approach is to store the mean of learned representations, thereby keeping the memory size equivalent to the number of seen individuals. We use ${\vec{V}}_{m}^{\left(t\right)}\in R^{c_{old}^{t}\times r}$ to denote current mean of learned representations for all seen individuals, where $c_{old}^{t}$ is the number of already seen individuals before round *t*. Then, we can obtain the objective loss as follows:

(3) $$\begin{array}{c} O_{3}=\eta {∥{\vec{S}}^{\left(t\right)}-{\vec{V}}_{m}^{\left(t\right)}{\vec{V}}^{(t)⊤}∥}_{F}^{2}, \end{array}$$

where ${\vec{S}}^{\left(t\right)}\in {\left\{0,1\right\}}^{c_{old}^{t}\times n_{t}}$ is the similarity matrix between the means of seen individuals’ latent representations and the to-be-learned representations of new samples ${\vec{V}}^{\left(t\right)}$. The element 0 in the matrix ${\vec{S}}^{\left(t\right)}$ indicates that a particular mean and a sample do not correspond to the same individual, whereas an entry of 1 indicates that they indeed belong to the same individual.

Our memory needs to adapt and change accordingly as streaming data arrive. These changes can be categorized into two scenarios: (1) If new individuals arrive, our memory needs to be expanded to store the means of those unseen individuals’ learned representations. In other words, this scenario can be viewed as the class-incremental case. (2) If new samples of previously seen individuals arrive with streaming data, our memory does not need to change its size, but it does require an update of the stored means of the existing individuals.

### 3.5. Overall Objective Function

By combining (1)–(3), the overall objective function of our method can be written as follows:

(4) $$\begin{array}{c} \min_{W^{\left(t\right)},G^{\left(t\right)},{\vec{R}}^{\left(t\right)},{\vec{V}}^{\left(t\right)}}O_{1}+O_{2}+O_{3}+\delta Re\left(W^{\left(t\right)},G^{\left(t\right)}\right), \end{array}$$

where δ is the trade-off parameter and Re(·) represents regularization terms. By optimizing this equation, we can obtain the representations ${\vec{V}}^{\left(t\right)}$ for samples at round *t*.

### 3.6. Online Optimization

We propose an online iterative optimization algorithm for (4). Our algorithm requires several iterations in order to converge, and each iteration has four sub-problems, in order to learn all variables. Specifically, within each sub-problem, we optimize one variable while keeping the others fixed. The details of one iteration at the *t*-th round are shown below.

**Step 1: Updating $W^{\left(t\right)}$.** We omit terms which are irrelevant with $W^{\left(t\right)}$ in (4) and present the sub-problem of $W^{\left(t\right)}$, as follows:

(5) $$\begin{array}{c} \min_{W^{\left(t\right)}}\;\;\;\alpha {∥{\tilde{V}}^{\left(t\right)}-{\tilde{X}}^{(t)⊤}W^{\left(t\right)}∥}_{F}^{2}+\\ \;\;\;\;\alpha {∥{\vec{V}}^{\left(t\right)}-{\vec{X}}^{(t)⊤}W^{\left(t\right)}∥}_{F}^{2}+\delta {∥W^{\left(t\right)}∥}_{F}^{2}, \end{array}$$

We can easily obtain the solution for the problem above by setting its gradient, with respect to $W^{\left(t\right)}$, to zero, as follows:

(6) $$\begin{array}{c} W^{\left(t\right)}={\left(C_{1}^{\left(t\right)}+\frac{\delta }{\alpha }I\right)}^{-1}C_{2}^{\left(t\right)}, \end{array}$$

where I denotes an identity matrix, $C_{1}^{\left(t\right)}={\tilde{X}}^{\left(t\right)}{\tilde{X}}^{(t)⊤}+{\vec{X}}^{\left(t\right)}{\vec{X}}^{(t)⊤}$, and $C_{2}^{\left(t\right)}={\tilde{X}}^{\left(t\right)}{\tilde{V}}^{\left(t\right)}+{\vec{X}}^{\left(t\right)}{\vec{V}}^{\left(t\right)}$. Furthermore, we can reformulate $C_{1}^{\left(t\right)}$ into the following scheme:

(7) $$\begin{array}{c} \begin{array}{cc} & C_{1}^{\left(t\right)}={\tilde{X}}^{\left(t\right)}{\tilde{X}}^{(t)⊤}+{\vec{X}}^{\left(t\right)}{\vec{X}}^{(t)⊤}\\ & \;\;\;\;\;\;\;=\left[{\tilde{X}}^{\left(t-1\right)};{\vec{X}}^{\left(t-1\right)}\right]{\left[{\tilde{X}}^{\left(t-1\right)};{\vec{X}}^{\left(t-1\right)}\right]}^{⊤}+{\vec{X}}^{\left(t\right)}{\vec{X}}^{(t)⊤}\\ & \;\;\;\;\;\;\;=C_{1}^{\left(t-1\right)}+{\vec{X}}^{\left(t\right)}{\vec{X}}^{(t)⊤}. \end{array} \end{array}$$

From (7), it is obvious that $C_{1}^{\left(t\right)}$ is composed with two terms. The first term $C_{1}^{\left(t-1\right)}$ does not need to be computed in the current round, as we already obtained its value during the last round. The second term needs to be calculated, but it is only associated with the data in the current round. Thus, (7) can be efficiently obtained. Similarly, we obtain $C_{2}^{\left(t\right)}=C_{2}^{\left(t-1\right)}+{\vec{X}}^{\left(t\right)}{\vec{V}}^{\left(t\right)}$.

Our design has two major advantages. (1) When learning $W^{\left(t\right)}$, knowledge learned during former rounds can be preserved and used. (2) $W^{\left(t\right)}$ is incrementally learned, making the optimization of this sub-problem efficient.

**Step 2: Updating $G^{\left(t\right)}$.** Analogous to $W^{\left(t\right)}$, the closed-form solution of $G^{\left(t\right)}$ can be given as follows:

(8) $$\begin{array}{c} G^{\left(t\right)}=C_{2}^{\left(t\right)}{\left(C_{3}^{\left(t\right)}+\frac{\delta }{\beta }I\right)}^{-1}, \end{array}$$

where $C_{3}^{\left(t\right)}=C_{3}^{\left(t-1\right)}+{\vec{V}}^{(t)⊤}{\vec{V}}^{\left(t\right)}$. Notably, we can temporarily store the value of $C_{3}^{\left(t-1\right)}$ from the last round and directly use it to obtain the variable $C_{3}^{\left(t\right)}$ during the current round, which ensures that the online optimization is extremely efficient.

**Step 3: Updating ${\vec{R}}^{\left(t\right)}$.** When fixing other variables and omitting irrelevant terms, the objective function used to solve ${\vec{R}}^{\left(t\right)}$ can be rewritten as follows:

(9) $$\begin{array}{c} \min_{{\vec{R}}^{\left(t\right)}}\;\;\theta {∥{\vec{R}}^{\left(t\right)}-{\vec{V}}^{\left(t\right)}∥}_{F}^{2}+\gamma {∥{\vec{A}}^{\left(t\right)}-{\vec{H}}^{\left(t\right)}{\vec{R}}^{(t)⊤}∥}_{F}^{2}. \end{array}$$

We can also set the derivative of this sub-problem, with respect to ${\vec{R}}^{\left(t\right)}$, to zero. Then, the closed-form solution of ${\vec{R}}^{\left(t\right)}$ is as follows:

(10) $$\begin{array}{c} {\vec{R}}^{\left(t\right)}=\left(\theta {\vec{V}}^{\left(t\right)}+\gamma {\vec{A}}^{(t)⊤}{\vec{H}}^{\left(t\right)}\right){\left(\theta I+\gamma {\vec{H}}^{(t)⊤}{\vec{H}}^{\left(t\right)}\right)}^{-1}. \end{array}$$

**Step 4: Updating ${\vec{V}}^{\left(t\right)}$.** Analogous to others, the objective function used to solve ${\vec{V}}^{\left(t\right)}$ can be reformulated as follows:

(11) $$\begin{array}{c} \begin{array}{cc} & \min_{{\vec{V}}^{\left(t\right)}}\;\;\beta {∥{\vec{X}}^{\left(t\right)}-G^{\left(t\right)}{\vec{V}}^{(t)⊤}∥}_{F}^{2}+\alpha {∥{\vec{V}}^{\left(t\right)}-{\vec{X}}^{(t)⊤}W^{\left(t\right)}∥}_{F}^{2}\\ & +\theta {∥{\vec{R}}^{\left(t\right)}-{\vec{V}}^{\left(t\right)}∥}_{F}^{2}+\eta {∥{\vec{S}}^{\left(t\right)}-{\vec{V}}_{m}^{\left(t\right)}{\vec{V}}^{(t)⊤}∥}_{F}^{2}. \end{array} \end{array}$$

Similarly, we can obtain the closed solution of ${\vec{V}}^{\left(t\right)}$, as follows:

(12) $$\begin{array}{c} \begin{array}{cc} & {\vec{V}}^{\left(t\right)}=(\alpha {\vec{X}}^{(t)⊤}W^{\left(t\right)}+\beta {\vec{X}}^{(t)⊤}G^{\left(t\right)}+\eta {\vec{S}}^{(t)⊤}{\vec{V}}_{m}^{\left(t\right)}+\theta \\ & {\vec{R}}^{\left(t\right)}){\left(\left(\alpha +\theta +\eta \right)I+\beta G^{(t)⊤}G^{\left(t\right)}+\eta {\vec{V}}_{m}^{(t)⊤}{\vec{V}}_{m}^{\left(t\right)}\right)}^{-1}. \end{array} \end{array}$$

**Overall Algorithm:** The above four steps constitute one whole iteration of our iterative online optimization algorithm. When training the model, we can update all the variables and repeat the process iteratively until the objective function converges. Algorithm 1 summarizes the proposed optimization.
**Algorithm 1: The online optimization of our method at round *t*.****Input**: the *t*-th data chunk with features ${\vec{X}}^{\left(t\right)}$; information stored in our memory ${\vec{V}}_{m}^{\left(t\right)}$; auxiliary variables $C_{1}^{\left(t-1\right)}$, $C_{2}^{\left(t-1\right)}$, and $C_{3}^{\left(t-1\right)}$; trade-off parameters; iteration number *T*.**Output**: Projection matrix $W^{\left(t\right)}$.**Procedure:**   Randomly initialize all variables $W^{\left(t\right)}$, $G^{\left(t\right)}$, ${\vec{R}}^{\left(t\right)}$, and ${\vec{V}}^{\left(t\right)}$;  **for** iter = 1,⋯,T **do**      Updating $W^{\left(t\right)}$ with (6);      Updating $G^{\left(t\right)}$ with (8);      Updating ${\vec{R}}^{\left(t\right)}$ with (10);      Updating ${\vec{V}}^{\left(t\right)}$ with (12);   **end for****Return**: auxiliary variables $C_{1}^{\left(t\right)}$, $C_{2}^{\left(t\right)}$, and $C_{3}^{\left(t\right)}$; variables $W^{\left(t\right)}$, $G^{\left(t\right)}$, ${\vec{R}}^{\left(t\right)}$, and ${\vec{V}}^{\left(t\right)}$.

### 3.7. Convergence Proof

To theoretically comprehend our method, we prove the convergence of the proposed alternating iterative algorithm for objective function optimization as follows. Let $L(W^{\left(t\right)},G^{\left(t\right)},{\vec{R}}^{\left(t\right)},{\vec{V}}^{\left(t\right)})$ denote the entire objective function in (4) at the *t*-th data chunk. As previously shown, there exists a closed-form solution for each variable in the corresponding sub-problem, and we obtain $L(W_{T+1}^{\left(t\right)},G_{T+1}^{\left(t\right)},{{\vec{R}}^{\left(t\right)}}_{T+1},{\vec{V}}_{T+1}^{\left(t\right)})$⩽$L(W_{T+1}^{\left(t\right)},G_{T+1}^{\left(t\right)},{{\vec{R}}^{\left(t\right)}}_{T+1},{\vec{V}}^{\left(t\right)})$⩽$L(W_{T+1}^{\left(t\right)},G_{T+1}^{\left(t\right)},{\vec{R}}^{\left(t\right)},{\vec{V}}^{\left(t\right)})$⩽$L(W_{T+1}^{\left(t\right)},G^{\left(t\right)},{\vec{R}}^{\left(t\right)},{\vec{V}}^{\left(t\right)})$⩽$L(W^{\left(t\right)},G^{\left(t\right)},{\vec{R}}^{\left(t\right)},{\vec{V}}^{\left(t\right)})$, where *T* is the iterative round. The objective function is a summation of positive norms and the objective loss monotonously decreases in each iteration, where $L(W^{\left(t\right)},G^{\left(t\right)},{\vec{R}}^{\left(t\right)},{\vec{V}}^{\left(t\right)})$ is bounded below due to its four positive terms. According to the bounded monotone convergence theorem [37], our method will converge to a local optimal solution.

### 3.8. Matching

After training the model, we obtain the matrix $W^{\left(t\right)}$, which can project the extracted ECG features into the learned representations. When matching, we use $X_{query}$ and $X_{registered}$ to denote the extracted ECG features of query samples and registered samples, respectively. Then, matching can be conducted based on representations of the samples $X_{query}^{⊤}W^{\left(t\right)}$ and $X_{registered}^{⊤}W^{\left(t\right)}$. Specifically, if the Euclidean distance between one query’s representation and one registration’s representation of an individual is the smallest distance possible, the query is considered to belong to this individual.

## 4. Experiments

### 4.1. Experimental Settings

#### 4.1.1. Datasets

We used two popular datasets to conduct the experiments, i.e., **MIT-BIH** [38] and **CYBHiDB** [39]. For the MIT-BIH dataset, the acquisition setting is on-the-person [40], which typically involves the use of multiple electrodes that are attached to the skin surface. This dataset contains 47 individuals, corresponding to 48 two-channel ambulatory ECG recordings. The second dataset, CYBHiDB, is one of the more challenging off-the-person datasets, and its data are acquired from hand palms and fingertips. Following the experimental settings used in existing works, we adopted a long-term approach with 63 healthy participants, including two distinct sessions separated by three months. Specifically, we defined these two sessions as T1 and T2 in the subsequent sections. For both datasets, we randomly selected five homogenous samples for each individual to construct the test sets, while we left the other samples to form the training sets.

#### 4.1.2. Evaluation Metrics

Two tasks were used for evaluation, i.e., identification and verification. In the identification mode, we used accuracy as an evaluation metric, representing the percentage of correctly identified individual heartbeat signals. For the authentication mode, we computed a similarity score between one heartbeat and all other heartbeats within the same database. The equal error rate (EER) denotes the point where the false acceptance rate (FAR) and false rejection rate (FRR) are equal within a specified threshold. Higher accuracy values signify superior performance, and lower EER values indicate better performance.

#### 4.1.3. Extracting Features from ECG Signals

As stated above, our method takes base features ${\vec{X}}^{\left(t\right)}$ as inputs at round *t*. In order to obtain ${\vec{X}}^{\left(t\right)}$, we firstly processed the ECG signals to obtain the heartbeat data, which are segmented by multiple sampling points on both sides of the R peak detected using the Pan–Tompkin algorithm [41]. One heartbeat corresponds to 260 sampling points in the MIT-BIH dataset and 600 sampling points in the CYBHiDB dataset, respectively. Then, we extracted 1DMRLBP features [42] to serve as the input ${\vec{X}}^{\left(t\right)}$.

#### 4.1.4. Online Setting and Implementation Details

As our method is designed for application in an online setting and with incremental data, we strictly followed the settings used in an existing work [17]. Specifically, the MIT-BIH dataset includes five data chunks, with the first four chunks containing 70 samples and the last round carrying the remaining 49 samples. The CYBHiDB dataset has six rounds. The first five rounds contain 108 samples, while the sixth chunk contains 27 samples. The Hadamard matrix is defined as $2^{7}\times 2^{7}$. We set the dimensionality of the latent representation space, *r*, to 128. The iteration number *T* was six. For the trade-off parameters, most of them were set to 1 except for δ=0.01, based on our parameter-sensitive analysis.

### 4.2. Comparison with the State-of-the-Art Method

We conducted comparisons with the state-of-the-art baseline method on two datasets. The experimental results for the MIT-BIH dataset are shown in Table 1, and the results for the CYBHiDB dataset are shown in both Table 2 and Table 3. As CYBHiDB contains T1 and T2 sessions, we reported two kinds of experimental results: within-session and across-session, which depend on whether the training data and testing data belonged to the same session. Within-session means that the training and testing data came from the same session, while across-session trains models on one session and tests models on the other session. The results of the traditional baseline methods on the two datasets were obtained via batch-based training. The results for our method and the online baseline method [17] were obtained by training the models in the online mode.

After analyzing the tables, we made the following observations: (1) In most cases, our proposed method offered the best performance, demonstrating its effectiveness. (2) When comparing our method with the online baseline method, we found that our method performed better, indicating that it can better alleviate the catastrophic forgetting problem. (3) Our method achieved a more satisfying performance than deep learning-based methods [11,12,35,43,44,47]. (4) Most baseline methods are traditional methods and they are trained in batch mode, while our method is trained using several data chunks, one by one. Although achieving better results through batch-based training is theoretically simpler than using online methods, our online approach still offers a superior performance. This phenomenon indicates that our designed online training model is also capable of fully utilizing all streaming data, showing that our method is a more practical solution for real-world applications. In summary, our method has shown promising results on both datasets, outperforming all baseline methods and demonstrating its effectiveness in handling streaming data in an online learning setting.

### 4.3. Further Analysis

#### 4.3.1. Ablation Study

We designed ablation experiments to assess the effectiveness of our method’s main components, and the results are outlined in Table 4. OURS_BR represents a variant that retains the prototype learning (PL) and memory enhancement (ME) modules while removing the bidirectional regressions (BR) module. OURS_PL denotes a variant with the BR and ME modules retained but without the prototype learning module. The OURS_ME variant preserves the BR and PL modules while discarding the memory enhancement module. As can be seen in Table 4, our method demonstrates a superior performance to all three variants. The experimental results emphasize the significance of bidirectional regressions, individuals’ prototype learning, and memory enhancement modules in our proposed online ECG biometrics approach.

#### 4.3.2. Parameters Sensitive and Convergence Analysis

**Figure 3:**
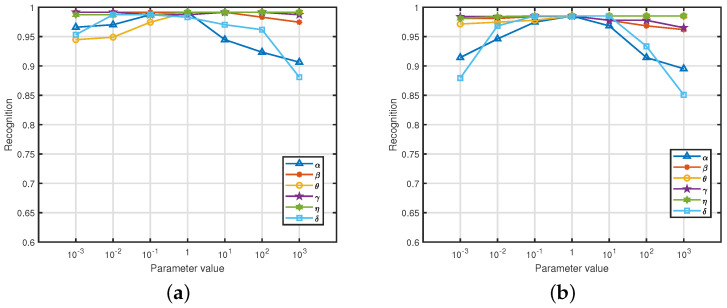
Subfigures (**a**,**b**) show the results of the parameter-sensitive analysis of the MIT-BIH and CYBHiDB-T1 datasets, respectively.

In this section, we performed experiments to evaluate the sensitivity of the parameters. Our method involves several key parameters: (1) α and β control the weights of the bidirectional regressions module; (2) γ and θ control the module for prototypes learning for individuals; (3) η denotes the weight of the memory enhancement module; (4) δ is the trade-off parameter for the regularization term. We varied all parameters within the range of [0.001,1000] and plotted the experimental results from the two datasets in Figure 3. We can observe from Figure 3 that the performance of our method is not sensitive to differing values for most parameters. Hence, we set δ=0.01 and the values of other parameters to 1. Considering that the parameters of our model are insensitive to their values, our approach is easy to implement in real applications without the large costs of tuning parameters.

**Figure 4:**
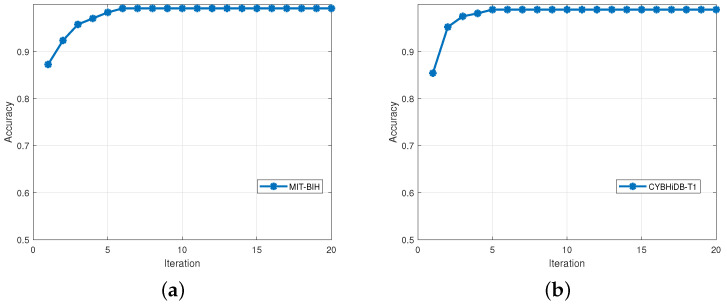
Subfigures (**a**,**b**) show the results of the convergence analysis of the MIT-BIH and CYBHiDB-T1 datasets, respectively.

We also conducted further experiments on the MIT-BIH and CYBHiDB-T1 datasets to validate the convergence of our method, and the results are shown in Figure 4. As shown in Figure 4, the accuracy improves steadily with increasing iterations, and the algorithm always converges within six iterations for the two datasets, confirming its convergence and efficiency.

#### 4.3.3. Streaming Data Handling Performance

**Figure 5:**
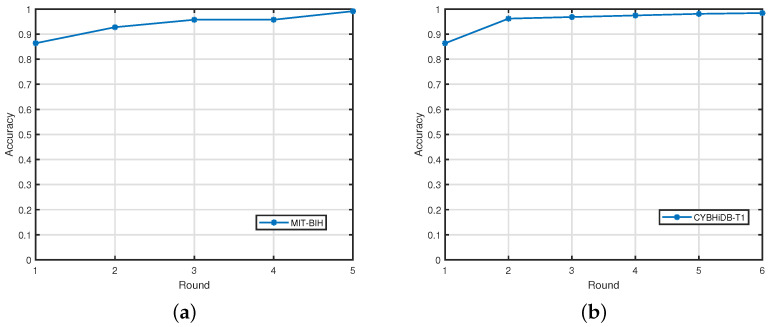
Subfigures (**a**,**b**) show the accuracy-round curves for the MIT-BIH and CYBHiDB-T1 datasets, respectively.

To comprehensively demonstrate the online performance of our model as streaming data arrive, we plotted the accuracy results, as new data chunks are incorporated into our method, on two datasets in Figure 5. As shown in Figure 5, with an increasing round count, the accuracy results improve steadily, and we can conclude that our method handled the streaming data well, offering a satisfying performance.

## 5. Conclusions and Future Works

In this paper, we proposed a new online method for ECG biometrics. Specifically, our model has three main modules and an online optimization algorithm. By using bidirectional regressions, prototypes learning, and memory enhancement modules, we can learn discriminative representations for samples from the extracted original ECG features. Due to the optimization algorithm, our method can be learned efficiently and incrementally. Experimental results on two benchmark datasets demonstrate the effectiveness of our method. Inspired by the success of deep learning and pre-trained models in various domains, we plan to explore their integration with online ECG biometrics to further enhance recognition performance and adaptability in the future.

## Figures and Tables

**Table 1:** Results from the MIT-BIH dataset.

Dataset	Method	Mode	EER (%)	Accuracy (%)
MIT-BIH	[11]	Batch	-	97.96
	[12]	Batch	-	98.57
	[28]	Batch	2.73	94.68
	[43]	Batch	-	96.5
	[44]	Batch	1.37	99.08
	[45]	Batch	-	97.66
	[46]	Batch	1.06	99.1
	[35]	Batch	-	98.0
	[17]	Online	0.64	99.15
	OURS	Online	0.62	99.25

**Table 2:** Within-session results for the CYBHiDB dataset.

Dataset	Method	Mode	EER (%)		Accuracy (%)	
			**T1**	**T2**	**T1**	**T2**
CYBHiDB	[4]	Batch	1.26	2.28	97.43	95.32
	[47]	Batch	1.85	3.35	97.12	94.95
	[48]	Batch	2.52	3.89	96.07	94.23
	[49]	Batch	5.45	6.53	93.52	91.41
	[46]	Batch	3.17	3.70	98.4	96.8
	[17]	Online	1.58	1.71	98.73	97.78
	Ours	Online	1.31	1.65	98.89	97.92

**Table 3:** Across-session results of the CYBHiDB dataset.

Method	Mode	Training	Testing	EER (%)	Accuracy (%)
[4]	Batch	T1	T2	10.26	87.75
		T2	T1	11.14	86.24
[47]	Batch	T1	T2	12.78	85.46
		T2	T1	12.83	84.46
[48]	Batch	T1	T2	13.87	84.35
		T2	T1	14.56	83.92
[49]	Batch	T1	T2	15.23	82.49
		T2	T1	14.78	83.83
[46]	Batch	T1	T2	6.17	92.86
		T2	T1	5.86	96.03
[17]	Online	T1	T2	3.17	96.51
		T2	T1	2.70	96.19
Ours	Online	T1	T2	2.77	95.56
		T2	T1	2.56	96.67

**Table 4:** Results of ablation study on the MIT-BIH and CYBHiDB datasets.

Variant	BR	PL	ME	MIT-BIH	CYBHiDB-T1	CYBHiDB-T2
OURS_BR	X	✓	✓	87.23	86.35	85.40
OURS_PL	✓	X	✓	86.38	85.71	85.08
OURS_ME	✓	✓	X	91.49	87.94	84.13
OURS	✓	✓	✓	99.25	98.89	97.92

## Data Availability

Data are contained within the article.
